# Biocompatible Gadolinium Oxide Nanoparticles Incorporated Doxorubicin Enables Magnetic Resonance and Photoacoustic Dual Imaging for Cancer Theranostics

**DOI:** 10.3390/nano16060343

**Published:** 2026-03-10

**Authors:** Xingchen Wang, Yuta Imai, Yu Kimura, Risako Miura, Hirohiko Imai, Teruyuki Kondo

**Affiliations:** 1 Department of Energy and Hydrocarbon Chemistry, Graduate School of Engineering, Kyoto University, Kyoto 6158510, Japan; 2 Innovation Research Center for Quantum Medicine, Gifu University School of Medicine, Gifu 501-1194, Japan

**Keywords:** theranostics, gelatin, gadolinium oxide, contrast agents, magnetic resonance imaging, photoacoustic imaging, doxorubicin

## Abstract

The engineering of theranostic nanoparticles, which integrate diagnostics and therapy in a single administration, enables targeted drug delivery and disease visualization. In cancer theranostics, gadolinium-based nanoparticles are valuable tools for noninvasive magnetic resonance imaging (MRI) and provide high-resolution images of the tumor. When MRI is combined with other imaging modalities, complementary therapeutic information is obtained for more accurate identification of tumor characteristics and precise guidance of anticancer drug delivery. Among the many possible modalities combined with MRI, photoacoustic imaging (PAI) is a candidate that enables sensitive in vivo detection of tumors. We have already succeeded in synthesizing biocompatible gelatin-coated gadolinium oxide nanoparticles with a controlled size by adjusting the timing of gelatin addition, which were a highly efficient contrast agent for MR and PA dual imaging. Herein, we conjugated a clinically used anticancer drug (doxorubicin, DOX) to size-defined and biocompatible gadolinium oxide nanoparticles which are novel theranostic probes. Succinylated gelatin enabled the electrostatic conjugation of DOX with gadolinium oxide nanoparticles, and the release of DOX was controlled through the enzymatic degradation of gelatin by matrix metalloproteinases-2 and -9 (MMP-2 and MMP-9), which are highly expressed in cancer cells. The released DOX efficiently inhibited the growth of HeLa cells in vitro and the growth of the inoculated tumor tissues in vivo. The dual-modality MRI and PAI capabilities provide anatomical information that assists in the localization and targeting of theranostic probes.

## 1. Introduction

In the last decade the concept of “Theranostics”, which combines therapeutic and diagnostic agents simultaneously in a single probe, has attracted much attention in oncology [1,2,3,4]. After historical achievements in the treatment and imaging of thyroid cancer [5], some radioactive agents have been clinically approved for theranostics for both the imaging and treatment of neuroendocrine tumors and prostate cancers [6,7,8]. Although these “radio”-theranostics [9], are quite useful for delivering a high dose of radiation directly to cancer cells, there are serious drawbacks, such as constant radiation emission with damage to healthy tissue surrounding the target, and low spatial resolution on PET/SPECT with difficulties in determining the precise location of the radiation source [10,11]. To overcome these limitations, other methodologies for theranostics, such as antibody–drug conjugates (ADCs) or nanoparticles NPs-based drug delivery systems (DDS), have been developed for treatment [12,13]. ADCs consist of recombinant monoclonal antibodies and/or their sub-components covalently bound to cytotoxic chemicals and imaging agents such as fluorophores. However, the use of ADCs is also limited by the inconsistent expression of target antigens, antibody immunogenicity, and adverse reactions such as the deconjugation of antibodies and drugs [14,15]. On the other side, NPs-based DDS has also been actively studied for cancer theranostics in the past decade [16,17]. The inherit size (10–100 nm) matched the length scales of tumor endothelial junctions and prevented reticuloendothelial entrapment. Thus, the enhanced permeability and retention (EPR) [18,19] of NPs also improved drug localization [19]. Nevertheless, the colloidal instability of NPs is a major problem for their clinical use, and there are many studies on the surface modification of NPs by biocompatible hydrophilic polymers [20,21,22,23,24,25,26].

Various anticancer reagents for theranostics have been conjugated to NPs [27,28]. The most widely used reagent is doxorubicin hydrochloride (DOX) [25,29,30], which acts synergistically via several mechanisms. The most important role is its intercalation into DNA and the inhibition of type II topoisomerase, eventually impeding DNA resealing and disrupting DNA replication and transcription in cancer cells, leading to their death [31,32]. Another important role is the generation of free radicals, leading to DNA damage or lipid peroxidation [31,33]. Also, as physical properties, DOX has cationic properties and hydrophobicity because of its amine group and anthraquinone structure. In the previous reports, DOX-PEG-PLA nanoparticles with hydrophobic interaction between DOX and PLA [34], DOX-Gelatin-Fe nanoparticles with electrostatic and hydrophobic interaction with DOX and gelatin [35], and chemically bonded PEGylated DOX-gelatin [36] between DOX and gelatin have been synthesized, and their functions evaluated as anticancer reagents. These reports implied that DOX could be interacted with gelatin through hydrophobic and electrostatic interaction. In fact, DOX can be conjugated with biocompatible peptides such as gelatin through electrostatic and hydrophobic interactions for the improvement of drug penetration into the targeted cancer tissues, and to avoid free DOX-induced cardiotoxicity [26]. Moreover, gelatin can be effectively degraded by gelatinases, including matrix metalloproteinases-2 and 9 (MMP-2 and -9), which are overexpressed in tumor sites during angiogenesis and tumor metastasis [37]. It was reported that gelatin-conjugated DOX was effective in targeting delivery to tumor tissues, minimizing distribution in normal tissues, especially the myocardium, and reducing the administration dose leading to improvements in their therapeutic efficacy at reducing the side effects [38].

In terms of diagnosis, apart from nuclear imaging, magnetic resonance imaging (MRI) provides high spatial resolution without depth limitation, while the sensitivity is relatively low [39,40]. In contrast, photoacoustic imaging (PAI) has the advantages of optical and acoustic methods, and PAI is a novel non-invasive and non-ionizing imaging modality with high sensitivity and resolution but is limited in path-length to approximately 7 cm [41,42,43,44]. Accordingly, the combination of MRI with PAI overcomes the individual limitations by synergic effect. This combination can assist in the localization and targeting of photoacoustic probes using high-resolution images and anatomical information from MRI. Our group have developed nanoparticulate probes for dual MR and PA imaging [39,45], and theranostic probes consisting of the MRI and PAI contrast agents were reported for the visualization and monitoring of the target tumor and their treatment [46].

In this study, DOX was immobilized to biocompatible succinylated PEG-gelatin (SPG)-modified gadolinium oxide (Gd_2_O_3_) nanoparticles as a potential MR–PA dual imaging probe. The reaction of succinic anhydride to gelatin generated additional carboxyl groups which enabled DOX to be interacted with gelatin electrostatically. The introduction of PEG would improve the aqueous dispersity of the nanoparticles. The SPG–DOX–Gd NPs were evaluated for the function of enzyme-responsive drug release, anticancer activity, and dual MR–PA imaging performance, which all supported the conclusion that they are an effective theranostic probe.

## 2. Materials and Methods

### 2.1. Materials

All reagents purchased from commercial suppliers were used without further purification, unless otherwise stated. Diethylene glycol (DEG, 99%), doxorubicin hydrochloride (DOX, 99%), acetone (99%), ethanol (99.5%), 2–[4–(2–hydroxylethyl)–1–piperazinyl] ethanesulfonic acid (HEPES, 99%), 2–[4–(2–hydroxylethyl)–1–piperazinyl] ethanesulfonic acid sodium salt (HEPES–Na, 99%), sodium dihydrogen phosphate dehydrate (99.0%), disodium hydrogen phosphate (99.0%), succinic anhydride (98%), Dulbecco’s phosphate-buffered saline (PBS), trypsin–EDTA solution (2.5 g/L trypsin, 1 mM EDTA), Cell Counting Reagent SF, and phosphoric acid (85%) were purchased from Nacalai Tesque Inc. (Kyoto, Japan). Gadolinium nitrate hexahydrate (Gd(NO_3_)_3_·6H_2_O, 99.5%), sodium hydroxide (97.0%), gadolinium standard solution (1000 ppm in 1 N HNO_3_, analytical grade), Dulbecco’s modified Eagle’s medium (DMEM; high glucose, containing L-glutamine, phenol red, and sodium pyruvate), and isoflurane (98.0%) were purchased from FUJIFILM Wako Pure Chemical Industries (Osaka, Japan). Acetonitrile (MeCN; ≥99.9% (GC)), recombinant human matrix metalloproteinase-2 (MMP-2, activated form, PF023), human recombinant tissue inhibitor of metalloproteinase-1 expressed in *E. coli* (TIMP-1, SRP3173), human recombinant tissue inhibitor of metalloproteinase-2 expressed in *E. coli* (TIMP-2, SRP3174), and penicillin (10,000 units/mL)–streptomycin (10 mg/mL) solution were purchased from Sigma–Aldrich Japan K.K. (Tokyo, Japan). Ultrapure water was prepared using a Millipore Direct–Q 3UV system (Millipore Inc., Billerica, MA, USA). Gelatin (Mw: 5000) isolated from porcine skin with an acidic process of collagen (Nitta Gelatin Co., Osaka, Japan) was kindly supplied from Professor Yasuhiko Tabata (Institute for Life and Medical Science, Kyoto University, Kyoto, Japan). Monomethoxy–*N*–hydroxysuccinimide–activated ester–polyethylene glycol (NHS–PEG, SUN BRIGHT ME-100HS, Mw: 10,000) was purchased from NOF Corporation (Tokyo, Japan). Fetal bovine serum (FBS) was purchased from Serana (Brandenburg, Germany). Medetomidine hydrochloride (Domitor^TM^ injection, Nippon Zenyaku Kogyo Co., Ltd., Fukushima, Japan), midazolam injection (Fuji Pharma Co., Ltd., Tokyo, Japan), and butorphanol tartrate (Vetorphale^TM^ injection, Meiji Animal Health Co., Ltd., Kumamoto, Japan) were used for animal experiments.

### 2.2. Synthesis of Succinylated PEG-Gelatin (SPG)

Gelatin (750 mg, 0.15 mmol, amine content: 0.375 mmol) was dissolved in 0.020 M phosphate buffer (pH 6.9, 50 mL) at r.t. with stirring for 12 h in a 100 mL recovery flask. NHS–PEG (500 mg, 0.05 mmol) was then added, and the mixture was stirred at r.t. for 12 h. Following purification by ultrafiltration (Amicon Ultra^TM^, Mw cutoff (MWCO): 10,000, MilliporeSigma, Burlington, MA, USA), succinic anhydride (150 mg, 1.5 mmol) was added at r.t. in 0.1 M HEPES buffer (pH 8.0, 25 mL). After conjugation for 12 h, succinylated PEG-gelatin (SPG) was purified by ultrafiltration (MWCO: 10,000) and lyophilized.

### 2.3. Synthesis of Gadolinium Oxide Nanoparticles (Gd_2_O_3_ NPs) Dispersed in Water

Gd_2_O_3_ NPs were synthesized according to a previously reported method with some modifications [45]. Gd(NO_3_)_3_·6H_2_O (451 mg, 1.0 mmol) was completely dissolved in DEG (1.0 mL, 13.6 mmol) by heating at 100 °C with stirring in a Schlenk flask. Then, NaOH pellets (50 mg, 1.25 mmol) and three drops of water were added, and the mixture was heated at 140 °C for 1 h, followed by further heating at 175 °C for 4 h. The color of the mixture gradually changed to dark brown. To isolate the nanoparticles, the above reaction mixture was cooled to room temperature and dropped into a large excess of acetone (40 mL) to precipitate Gd_2_O_3_ NPs. Gd_2_O_3_ NPs were isolated by centrifugation (6400× *g*, 10 min), and the precipitate was washed with acetone (40 mL, three times). The isolated Gd_2_O_3_ NPs were dispersed in ultrapure water.

### 2.4. Synthesis of Succinylated PEG-Gelatin-Modified Gd_2_O_3_ NPs (SPG–Gd NPs) and DOX-Incorporated SPG-Gd NPs (SPG–DOX–Gd NPs)

The dispersed Gd_2_O_3_ nanoparticles in water (Gd content: 20 mg/20 mL) were added to an aqueous solution of SPG (200 mg, 0.013 mmol) with stirring at r.t. for 12 h. After purification with ultrafiltration (Amicon Ultra^TM^, MWCO: 50,000, 7 times), SPG–Gd NPs were obtained.

SPG–DOX–Gd NPs were prepared using a method similar to that described above. Gd_2_O_3_ NPs dispersed in water were added to a mixed aqueous solution of SPG (200 mg, 0.013 mmol) with various amounts of DOX. After stirring and conjugation at r.t. for 12 h, SPG–DOX–Gd NPs were purified via ultrafiltration (MWCO: 50,000) using water as the wash medium until DOX was not observed in the wash liquid.

The concentration of Gd was determined by polarized Zeeman atomic absorption spectrometry (AAS; Z–2710, Hitachi Ltd., Tokyo, Japan) equipped with a hollow cathode lamp (L233-64NB, Hamamatsu Photonics K.K., Hamamatsu, Japan) using a Gd standard solution (1000 ppm, analytical grade, Fujifilm Wako, Osaka, Japan) at 407.9 nm. The hydrodynamic mean diameter of the nanoparticles dispersed in water was determined by dynamic light scattering (DLS; Zetasizer Nano ZS, Malvern Instruments, Worcestershire, UK). The surface electric potential of the nanoparticles (ζ potential) was determined by electrophoresis light scattering analysis (ELS; Zetasizer Nano ZS) with disposable capillary cell (DTS1061, sample loading: *ca*. 1.0 mL).

### 2.5. Quantification of Incorporated DOX into the SPG–DOX–Gd NPs

The amount of incorporated DOX was calculated using the following formula:

DOX*_incorporated_* = DOX*_prepared_* − sum of DOX*_filtrate_* in wash liquid by ultrafiltration

To calculate the amount of DOX in the ultrafiltration wash liquid (Amicon Ultra^TM^, MWCO: 50,000), the same amount (1 mL) of the wash liquid on the synthesis and trypsin–EDTA mixed solution were incubated with shaking at 1000 rpm for 1.5 h at 37 °C to degrade the surface-conjugated SPG with DOX. After incubation, a 100 μL aliquot of the mixture was subjected to UHPLC analysis using a Nexera X2 system (Shimadzu Corporation, Kyoto, Japan) [47]. The analyses were performed at 40 °C using a Luna Omega C18 LC Column (00B-4742-AN, 50 × 2.1 mm, Phenomenex, Torrance, CA, USA). Samples were analyzed with an eluent gradient of pH 3.0 phosphoric acid solution and MeCN at a continuous flow rate of 0.25 mL/min (MeCN/phosphoric acid gradients: 0.5–5 min: 15/85–95/5, 8–10 min: 95/5–100/0, 10.01–15 min 100/0–15/85). The eluents were monitored by UV absorbance at 480 nm wavelength.

### 2.6. In Vitro Characterization of Enzyme-Responsive DOX Release from SPG–DOX–Gd NPs

SPG–DOX–Gd NPs were dispersed in PBS with or without 30 nM MMP-2 and incubated with shaking (1000 rpm) at 37 °C for 72 h [48]. A 100 μL aliquot of the mixture was collected and added to 400 μL PBS at specific time points. The released DOX was collected via ultrafiltration (Amicon Ultra^®^, MWCO: 50,000) as the filtrate. The amount of DOX released from the NPs was determined by HPLC analysis.

### 2.7. Cytotoxicity Assay of SPG–DOX–Gd NPs

Human cervical adenocarcinoma cells (HeLa, BRC RCB3680) and mouse fibroblast cell lines (L929, ATCC CCL-1) were seeded on 96-multiwell cell culture plates (Corning Inc., Lowell, MA, USA) at a density of 1 × 10^4^ cells/cm^2^ and incubated at 37 °C under 5% CO_2_–95% air atmospheric pressure with saturated humidity. The culture medium (D–MEM/F–12 with 10% FBS, 100 U/mL penicillin, and 100 μg/mL streptomycin) was replaced with a fresh medium containing various concentrations of SPG–DOX–Gd NPs, SPG–Gd NPs, or DOX *alone* (*n* = 5) 24 h after cell seeding. After incubation for 48 h, the cell numbers were evaluated. After changing the fresh medium (90 μL), cell count reagent SF (10 μL) was added to each well, and the plates were incubated for 2 h at 37 °C. The absorbances at 450 and 600 nm were measured at 1 and 2 h using a spectrophotometer (iMark^TM^ Microplate reader, Bio Rad Laboratories Inc., Hercules, CA, USA). The absorbance was normalized to that of cells proliferating in culture medium without the reagents.

Inhibition of MMPs and the effect for DOX release from SPG–DOX–Gd NPs was evaluated under co-incubation with tissue inhibitor of metalloproteinases (the same amount mixture of TIMP-1 and TIMP-2) according to a previous report [39]. After incubation of HeLa cells (1 × 10^4^ cells/cm^2^) on 96-multiwell cell culture plates for 21 h, the cell culture medium was replaced with fresh medium (50 μL) containing 0–200 ng/mL TIMP-1 and TIMP-2 (*n* = 4). After incubation for 3 h, SPG–DOX–Gd NPs dispersion (DOX: 0.1 μg/mL; 50 μL) was added. After incubation for 16 h, cell counting was performed in the same manner.

### 2.8. Phantom MRI Acquisition and Proton Relaxivity Evaluation

Clinically used Gd–DTPA (Magnevist^TM^, gadopentetate dimeglumine; Bayer Holding Ltd., Tokyo, Japan) was used as a standard to evaluate the contrast-enhancing ability of the nanoparticles for MRI. MRI acquisition was carried out using a 7T small-animal MRI scanner at 20 °C (BioSpec 7T/20 USR equipped with a 72 mm inner diameter quadrature resonator; Bruker Corporation, Billerica, MA, USA) at the Medical Research Support Center of the Graduate School of Medicine, Kyoto University. Water dispersions of synthesized SPG–DOX–Gd NPs, SPG–Gd NPs, and Gd-DTPA (1.0, 0.50, 0.25, and 0.10 mM Gd) were prepared to calculate longitudinal and transverse relaxation time (*T*_1_ and *T*_2_). *T*_1_ was obtained by a rapid acquisition with a relaxation enhancement (RARE) sequence with repetition times (TR) of 5, 3, 1.5, 0.8, 0.4, 0.2, 0.1, and 0.05 s, echo time (TE)/effective TE = 11 ms, field of view (FOV) = 60 × 40 mm^2^, matrix size = 192 × 128, slice thickness = 2 mm, NA = 1, and RARE factor = 2. *T*_2_ was obtained using a multi-echo SE sequence (single slice) with TE = 10 ms, Effective TE = 10–320 ms with 10 ms steps (32 TEs), TR = 5000 ms, FOV = 60 × 40 mm^2^, matrix size = 192 × 128, slice thickness = 2 mm, and NA = 1. The longitudinal and transverse relaxivity coefficients (*r*_1_ and *r*_2_, L·mmol^−1^·s^−1^) were calculated from the slopes of the linear regression plots of Gd concentration versus the reciprocal of relaxation time ((1/*T_i_*) − (1/*T_i_*_solvent), where *i* = 1 for longitudinal and *i* = 2 for transverse relaxation).

### 2.9. In Vivo Tumor Reduction and Imaging Studies

All animal experiments were performed according to the Institutional Guidance of Kyoto University on Animal Experimentation. All animal experiments were approved by the Animal Experimentation Committee of the Faculty of Engineering, Kyoto University (Approval No.: 2022-03 and 2023-03). Tumor-bearing mice were prepared by breeding BALB/c nude mice (Slc-nu/nu, 6 weeks old, female, Japan SLC Inc., Shizuoka, Japan) for 10 days after the injection of HeLa cells (5.0 × 10^7^ cells with 50 uL Geltrex^TM^ LDEV-free reduced growth factor basement membrane matrix (Gibco A14132-02, Lot No.1019078B, Life Technologies, Grand Island, NY, USA), right back region) were anesthetized through the intraperitoneal administration of a mixture solution containing medetomidine hydrochloride (0.75 mg/kg), midazolam (4 mg/kg), and butorphanol tartrate (5 mg/kg) and the continuous inhalation of isoflurane (2–5%). This in vivo study was conducted in two independent experimental cycles. In each cycle, tumor-bearing mice were randomly divided into three groups (6 mice in each group), namely “SPG–DOX–Gd NPs”, “DOX *alone*”, and “PBS”. Data from the two cycles were combined for analysis (*n* = 12 mice per group). For the “SPG–DOX–Gd NPs” group, SPG–DOX–Gd NPs were administrated into mice twice a week via tail vein at 0.10 mmol Gd/kg and 10 mg DOX/kg (*n* = 12). The dose of Gd was the same as that of Gd-DTPA, and the dose of DOX was determined according to a previous report [49]. In contrast, for the “DOX *alone*” and the “PBS” groups, DOX solution (10 mg DOX/kg) and the same volume of PBS were injected into mice using the same schedule. On the same day as each administration, the size of the tumor tissue was measured manually using calipers, and body weight was recorded. The relative size of the tumor tissue was calculated as *V*/*V*_0_, where *V*_0_ and *V* represent the initial tumor volume before treatment and the tumor volume after treatment at a given time point, respectively. The body weights of the mice in each group were recorded during the treatment process.

In vivo *T*_1_-weighted MR images of anesthetized mice were obtained before and 2 h after the intravenous injection of nanoparticles using a spin echo sequence (TR/TE = 200/6.2 ms, FOV = 8 × 4 cm^2^, matrix size = 256 × 128, slice thickness = 2 mm, NA = 2) with a 7 T MRI system. The signal intensity (SI) of tumor was obtained from each MR image after standardizing by MATLAB software (R2025b Version 25.2.0.3042426), and the region of interest (ROI) was inside of respective tumors except for the outline of them with ImageJ (Version 1.54p). The SI-after administration divided by the SI-before administration was calculated as post/pre-SI enhancement.

In vivo PA images were obtained using a preclinical photoacoustic computed tomography scanner (Nexus 128, Endra Inc., Ann Arbor, MI, USA). Mouse images were obtained at the laser strength of 2.1 mJ/cm^2^ (710 nm, 20 Hz, 37 °C) under anesthesia with isoflurane. All images were displayed using the same color scale range (min–max: 750–2600, a.u.), and no per-image contrast adjustment was performed. This wavelength lies within the near-infrared window [50,51] and the UV-Vis-NIR absorbance spectra of SPG–DOX–Gd NPs show nonzero absorbance at 710 nm (Figure S1). To evaluate contrast enhancement on PA imaging, PA images were obtained at pre and 1 h after every administration on day 0 and day 11 via the tail vein (*n* = 12) (the first administration was performed on day 0). PA signals were evaluated as changes relative to the pre-administration images acquired under the same settings to reduce the influence of the endogenous hemoglobin background.

### 2.10. Statistical Analysis

Statistical evaluations were conducted using GraphPad Prism 10 (Version 10.6.1(799), GraphPad Software, La Jolla, CA, USA). Results are expressed as mean ± standard deviation (SD) from a minimum of three independent experiments (*n* ≥ 3) unless otherwise stated. Group differences were assessed using one-way analysis of variance (one-way ANOVA), followed by the Tukey–Kramer post hoc test for multiple-group comparisons. All tests were two-sided, and *p*-values below 0.05 were regarded as statistically significant.

## 3. Results and Discussion

### 3.1. The Physical Characterization of the Synthesized SPG–DOX–Gd NPs

Water-dispersed gadolinium oxide (Gd_2_O_3_) NPs were synthesized according to a modified method reported in the literature [45]. The isolated Gd_2_O_3_ NPs undergo coarse aggregation in pure water over the incubation time. Gelatin acts as a protective colloid that maintains size stability and enables diameter control from 20 to 200 nm by adjusting the timing of gelatin addition [45]. Conjugation of PEG to gelatin significantly improved the stability of Gd_2_O_3_ NPs in water, and treatment with succinic anhydride produced succinylated PEG-gelatin (SPG) with extra carboxyl groups on gelatin, which enabled the reaction with DOX. Subsequently, SPG–DOX–Gd NPs were prepared by mixing SPG–Gd NPs with various amounts of DOX. After the extensive washing of the reaction mixture with water, SPG–DOX–Gd NPs were isolated.

Based on our previous work [45] and other studies that used the same DEG-polyol synthetic route [52,53,54], we consider it reasonable that Gd_2_O_3_ nanoparticles prepared by this route can show an amorphous-like or poorly resolved XRD profile in the as-prepared state.

Dynamic light scattering (DLS) measurements showed that the hydrodynamic diameters of SPG–DOX–Gd NPs, SPG–Gd NPs, and Gd_2_O_3_ NPs dispersed in water were 24.32 ± 8.80, 26.61 ± 3.31, and 66.14 ± 4.77 nm after incubation for 3 d, respectively (Figure S1). Neither aggregation nor change in the mean diameters of SPG–DOX–Gd NPs and SPG–Gd NPs were observed, whereas Gd_2_O_3_ NPs were aggregated and deposited in water. These results indicate that SPG can serve as an efficient protective colloid for Gd_2_O_3_ NPs by increasing hydrophilicity and inhibiting aggregation.

The Figure S3 showed surface zeta potentials (ζ) of SPG–DOX–Gd NPs, SPG–Gd NPs, and Gd_2_O_3_ NPs were −1.38 ± 0.17, −4.47 ± 0.33, and +19.63 ± 0.57 mV, respectively. Surface modification of water-dispersed Gd_2_O_3_ NPs with SPG shifted the surface potential from positive to negative, which supports surface coverage by a SPG that exposes carboxyl groups at the interface. The hydrodynamic diameter increased after gelatin-based surface modification, which is consistent with the formation of a polymer layer on the particle surface.

### 3.2. Quantification of the Incorporated DOX in SPG–DOX–Gd NPs

**Figure 1:**
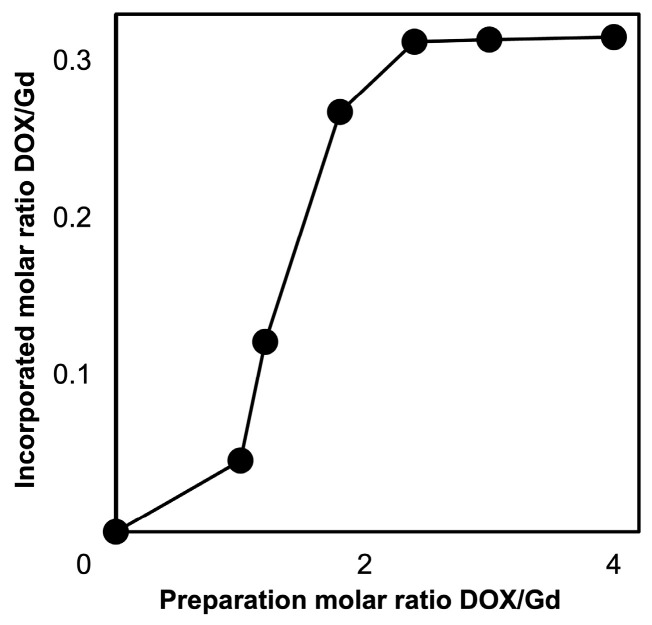
Incorporated amount of DOX into SPG–DOX–Gd NPs depending on various amounts of DOX in preparation.

To investigate the amount of DOX incorporated into the NPs, the progress of extensive wash with NPs by ultrafiltration (MWCO: 50,000 with ultrapure water at 37 °C) was evaluated using HPLC analysis. The results are summarized in Figure S3; no specific release of DOX from SPG–DOX–Gd NPs was observed after the third wash. As shown in Figure 1, the incorporated amount of DOX increased as the prepared amount of DOX increased until reaching a preparation molar ratio DOX/Gd of 2.4. The incorporation capacity of DOX to NPs was calculated by measuring the amount of DOX in the wash liquid using HPLC and was saturated at approximately 0.31 in the incorporated molar ratio DOX/Gd. This result proves the incorporated capacity of DOX is determined by the amount of carboxyl group on the SPG.

### 3.3. In Vitro Investigation of Enzyme-Responsive DOX Release from SPG–DOX–Gd NPs

**Figure 2:**
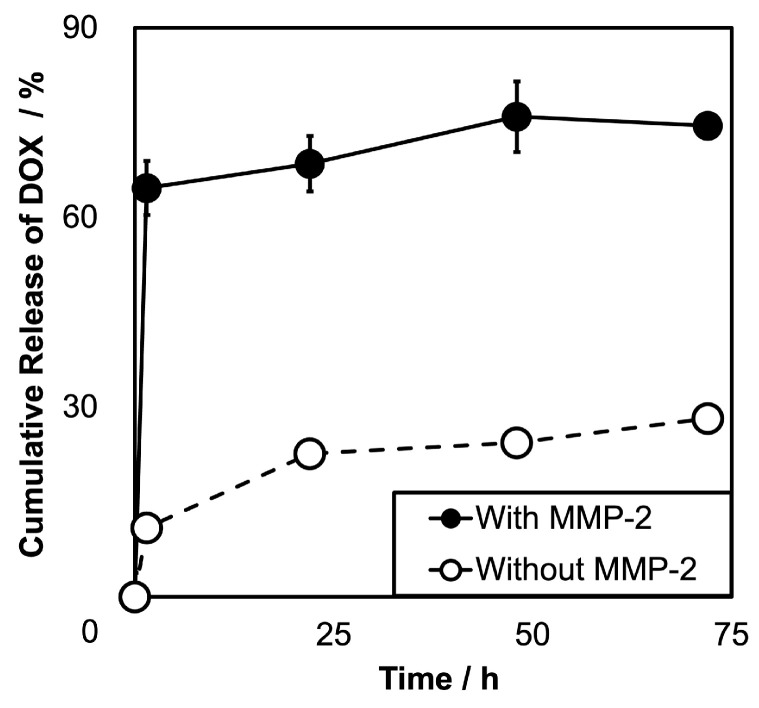
In vitro release of DOX from SPG–DOX–Gd NPs with (black circle) or without (white circle) MMP-2. Data are presented as mean ± SD, *n* = 3.

The release of DOX from SPG–DOX–Gd NPs was investigated in PBS solution in the presence and absence of 30 nM matrix metalloproteinase-2 (MMP-2) in vitro (Figure 2). In the absence of MMP-2, 10.87 ± 0.68% of the conjugated DOX in SPG–DOX–Gd NPs was released after the first 1.5 h, which is the nonspecific release of DOX. Even after 72 h in the absence of MMP-2, only 28.21 ± 2.15% of DOX was released. For comparison, Long et al. reported that DOX-loaded gelatin nanoparticles showed a release of about 70% in PBS (pH 7.4) by 48 h [55]. These results suggest that SPG–DOX–Gd NPs have excellent drug maintenance efficiency in a physiological environment. In contrast, 64.65 ± 3.75% of DOX was quickly released within 1.5 h in the presence of MMP-2, and after 72 h, 74.49 ± 5.59% of DOX was released. These results showed that SPG–DOX–Gd NPs have unusually high stability under the physiological environment, which inhibited the complete release (~100%) of DOX even in the presence of MMP-2, and some gelatin chains would remain on the surface of SPG–DOX–Gd NPs [37,56]. Accordingly, SPG–DOX–Gd NPs showed high stability, while the conjugated DOX was released from SPG–DOX–Gd NPs by an enzyme-responsive manner.

To further evaluate the stability of SPG–DOX–Gd NPs in serum-containing conditions, a release test was performed in PBS containing 10% fetal bovine serum (FBS) (pH 7.4) at 37 °C. The release of DOX was monitored from 0 to 72 h (*n* = 3) and quantified by HPLC after ultrafiltration (Amicon Ultra^®^, MWCO: 50,000) (Figure S5). The SPG–DOX–Gd NPs exhibited minimal DOX leakage, showing 19.45 ± 1.00% release at 72 h, which was comparable to the release in PBS alone. These results indicate that SPG–DOX–Gd NPs maintain good stability under physiologically relevant conditions in the presence of serum proteins.

The drug delivery system designed in this study can improve the targeting efficiency of the anticancer drug, DOX, by the EPR effect and persistent retention of the anticancer drug at cancer sites. As a result, the surface SPG of SPG–DOX–Gd NPs was degraded by MMP-2, which is highly expressed in tumor tissues, leading to the release of the chemotherapy drug.

### 3.4. In Vitro Cytotoxicity Assay

**Figure 3:**
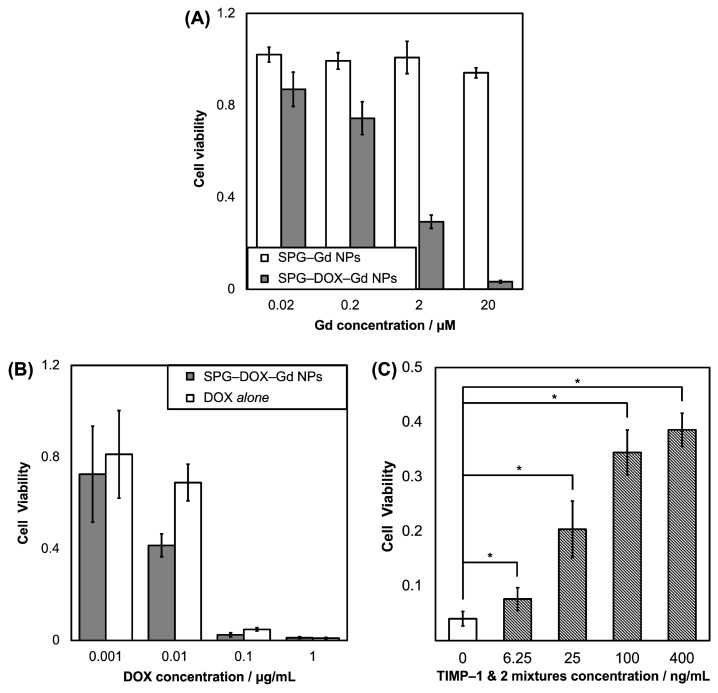
Cytotoxicity against HeLa cells by SPG–DOX–Gd NPs, SPG–Gd NPs (**A**), and DOX *alone* (**B**). Data are presented as mean ± SD, *n* = 5. (**C**): Cytotoxicity against HeLa cells by SPG–DOX–Gd NPs after preincubation with TIMPs mixture. Data are presented as mean ± SD, *n* = 4 and * *p* < 0.05.

The cytotoxicity of SPG–DOX–Gd NPs against HeLa cells, which express high levels of MMP-2 and -9 genes, was analyzed. The viability of HeLa cells was evaluated using the water-soluble tetrazolium salt (WST) assay, and the number of HeLa cells without samples was regarded as a control. Following the incubation of HeLa cells with SPG–DOX–Gd NPs, SPG–Gd NPs (Figure 3A) and DOX *alone* (Figure 3B) for 48 h, respectively, SPG–Gd NPs showed no cytotoxicity because the cell viability did not change regardless of the concentration. In contrast, HeLa cells exposed to SPG–DOX–Gd NPs or DOX *alone* died in a DOX concentration-dependent manner.

To verify the DOX released from SPG–DOX–Gd NPs related to MMP-2 and -9 expressed in HeLa cells, tissue inhibitor metalloproteinases (TIMP)-2 and -1 towards MMP-2 and/or -9, respectively, were pre-incubated with HeLa cells for 3 h before exposure to SPG–DOX–Gd NPs (Figure 3C). Cell viability increased with increasing concentrations of TIMP-1 and -2 mixtures. These results showed that the degradation of surface SPG of SPG–DOX–Gd NPs by MMP-2 and/or -9 was inhibited by TIMPs to suppress DOX release. These results indicated that the cytotoxicity of SPG–DOX–Gd NPs was derived from only DOX released from SPG–DOX–Gd NPs via the biodegradation of surface SPG by MMP-2 and/or -9, which proves that SPG–DOX–Gd NPs are a highly selective and MMP-2 and/or -9-driven anticancer agent for the tumor.

### 3.5. Evaluation of SPG–DOX–Gd NPs for an MRI Contrast Agent In Vitro

**Figure 4:**
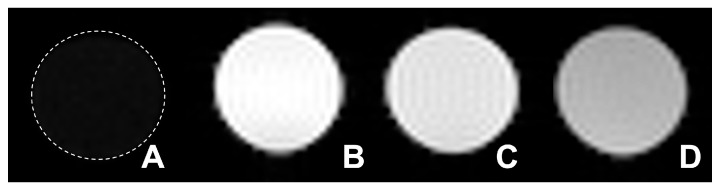
T1W phantom MR images of (**A**) water, (**B**) SPG–DOX–Gd NPs, (**C**) SPG–Gd NPs, and (**D**) Gd–DTPA (Magnevist^®^). The Gd concentration in the (**B**–**D**) image is 0.50 mM.

To investigate the potential of NPs as an MRI contrast agent, the longitudinal relaxation time *T*_1_ was measured, and the relaxivity *r*_1_ was estimated. Figure 4 shows *T*_1_-weighted (T1W) images of clinically used Gd–DTPA (Magnevist^®^) and the synthesized Gd NPs suspension in water (0.5 mM Gd). Two types of the synthesized Gd NPs showed brighter images than that of Gd-DTPA, and among them, SPG–DOX–Gd NPs gave the brightest T1W MR image. Notably, under the acquisition conditions used here, the SPG–DOX–Gd NPs did not show obvious signal darkening within the tested range of 0.10–1.0 mM Gd, which indicates that *T*_1_-positive contrast dominates within this tested concentration window.

The 1/*T*_1_ and 1/*T*_2_ values obtained from these images were plotted against the Gd concentration. Relaxivity was evaluated at Gd concentrations of 0.10, 0.25, 0.50, and 1.0 mM, and the linear regression showed excellent linearity within this range (coefficient of determination > 0.999). The slopes of the linear regression lines were used to calculate the relaxation values *r*_1_ and *r*_2_, as shown in Table 1. Although an *r*_2_*/r*_1_ ratio of 2.6 can be associated with *T*_2_-related signal quenching at sufficiently high local concentrations, this effect was not evident under our measurement conditions within 0.10–1.0 mM Gd. Additionally, several previous studies suggest that contrast agents with *r*_2_*/r*_1_ below 5 are generally regarded as *T*_1_ agents [57,58]. At higher local concentrations beyond the tested range, stronger *T*_2_-related signal loss may reduce the apparent *T*_1_-positive contrast. The highest *r*_1_ value of SPG–DOX–Gd NPs (15.4 mM^−1^s^−1^) may be attributed to their smaller hydrodynamic diameter. The smaller size increases the accessible surface area for water interaction around the Gd sites and can enhance longitudinal relaxation. Notably, this *r*_1_ value is higher than that typically reported of clinically approved Gd-based contrast agents, which show *r*_1_ values of about 3.6–6.3 mM^−1^s^−1^ [59]. These results clarified that SPG–DOX–Gd NPs have the potential to be an efficient positive contrast agent for MRI.

### 3.6. Evaluation of SPG–DOX–Gd NPs for Tumor Reduction In Vivo

**Figure 5:**
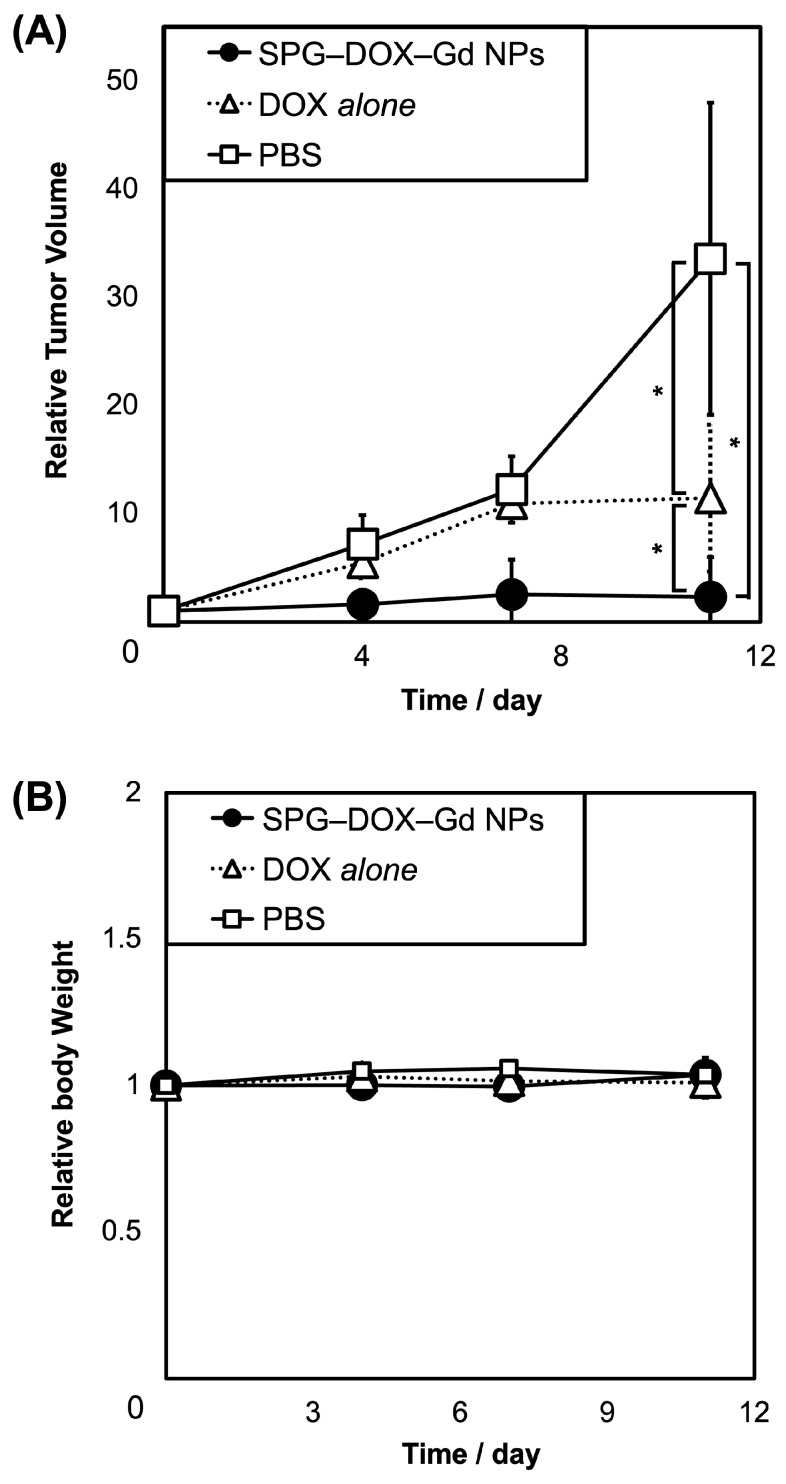
Time course of (**A**) tumor volume and (**B**) relative body weight of tumor-bearing mice in vivo with the administration of SPG–DOX–Gd NPs, DOX *alone*, and PBS solution. Data are presented as mean ± SD, *n* = 12 and * *p* < 0.05.

To investigate the potential of SPG–DOX–Gd NPs as an anticancer agent in vivo, the restraint of tumor growth was examined by the continuous manual monitoring of tumor volume. Mice were used to establish tumor-bearing models via the subcutaneous injection of HeLa cells. Treatment was initiated when the tumors reached a diameter of approximately 5 mm, which occurred approximately 10 days after inoculation. PBS solution (control), DOX *alone*, and SPG–DOX–Gd NPs were intravenously administered to HeLa cell-bearing mice on days 0, 4, 7, and 11, and tumor volumes were monitored for 11 days. The results clearly showed that the tumors grew rapidly in the PBS group, indicating that tumor growth was not affected by PBS solution (Figure 5). The tumors in mice administered with DOX *alone* grew slowly and then remained constant, indicating that DOX *alone* showed moderate antitumor efficacy. In contrast, the tumor volume of mice administered with SPG–DOX–Gd NPs showed much slower growth and eventually stopped. Considering all the results obtained, SPG–DOX–Gd NPs effectively accumulated at the tumor site by the EPR effect, and a pronounced anticancer effect was observed through the degradation of surface SPG of the NPs by MMP-2 and -9 at the tumor site.

### 3.7. Tumor Imaging After SPG–DOX–Gd NPs Administration by MRI and PAI

**Figure 6:**
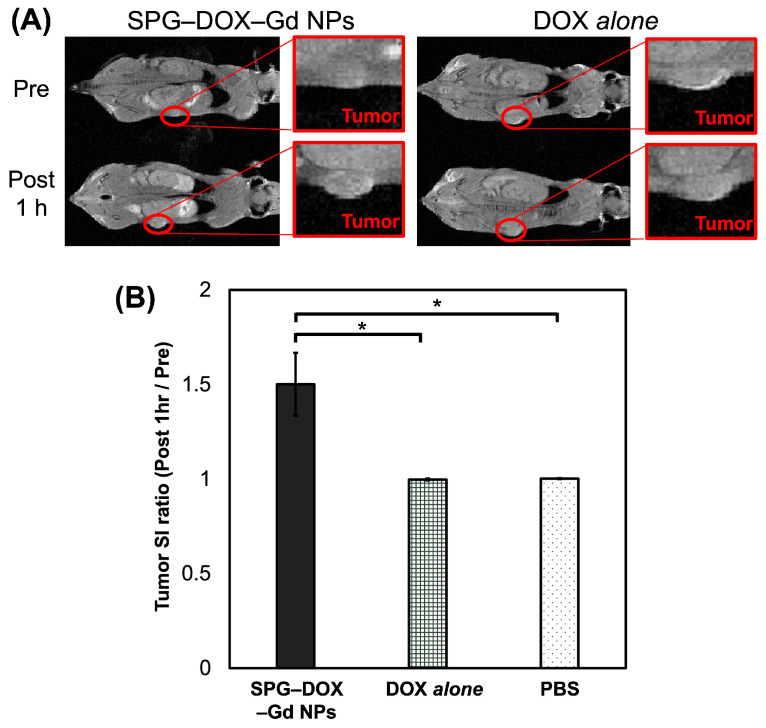
(**A**) The T1W MR images of HeLa cell-bearing mice at the administrated site of SPG–DOX–Gd NPs and DOX *alone*, respectively, before administration (pre) and 1 h after administration (post 1 h) at day zero. (**B**) The tumor SI ratios at the administrated sites of SPG–DOX–Gd NPs, DOX *alone*, and PBS, respectively (0.10 mmol Gd and 10 mg DOX/kg, intravenous administration, 7T, r.t.). Data are presented as mean ± SD, *n* = 12 and * *p* < 0.05.

To confirm the diagnostic ability of SPG–DOX–Gd NPs as a MR–PA dual imaging probe in vivo, the NPs were injected into HeLa cell-bearing mice via the tail vein. Figure 6A shows the T1W MR images of the mice before and 1 h after intravenous administration, and Figure 6B shows the tumor SI ratios at the tumor sites of SPG–DOX–Gd NPs, DOX *alone*, and PBS solution injected, respectively. At the tumor site administered with SPG–DOX–Gd NPs the contrast of the tumor site increased 1.50 times higher than those on average. No contrast enhancement was observed after the administration of DOX *alone* or PBS.

**Figure 7:**
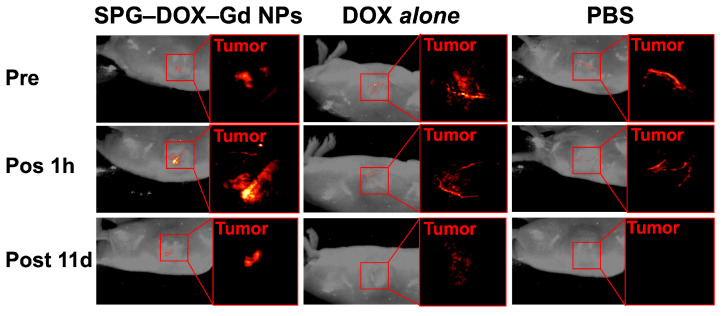
In vivo photoacoustic images of HeLa tumor-bearing mice acquired before administration (Day 0 Pre), at 1 h after intravenous administration on day 0 (Day 0 Post 1 h), and at 1 h after intravenous administration on day 11 (Day 11 Post 1 h). Mice received SPG–DOX–Gd NPs, DOX *alone*, or PBS. Imaging was performed at 710 nm with a repetition rate of 20 Hz. Doses were 0.1 mmol Gd/kg and 10 mg DOX/kg.

PA images were obtained before administration and 1 h after each twice-weekly administration, and the schedule is as follows: SPG–DOX–Gd NPs, DOX alone, and PBS solution were intravenously administrated on day zero (Day 0 Pre, Day 0 Post 1 h) and day 11 (Day 11 Post 1 h). Table 2 summarizes the SI ratios (post 1h/pre) derived from the MR and PA (Figure 7) images of the tumor site. Compared with representative nanoparticle reports, our SPG–DOX–Gd NPs show a favorable tumor-site SI response under the present imaging schedule [60,61]. As a result, both ratios after the administration of SPG–DOX–Gd NPs were higher than those after the administration of DOX alone and PBS solution at day zero. It was presumed that SPG–DOX–Gd NPs were accumulated in tumor tissues by the EPR effect to cause the enhancement of tumor site in both MR and PA images.

In this study, both in vitro and in vivo evaluations used HeLa models with high MMP-2 and -9 expression. A limitation of the present study is that the triggerable performance of SPG–DOX–Gd NPs depends on MMP activity, and the efficacy remains unclear in cancer models with low or uncertain MMP expression. In addition, comprehensive organ-specific safety evaluation and further optimization of imaging protocols will be important in future studies to support clinical translation.

## 4. Conclusions

We developed novel succinylated PEG-gelatin (SPG)-modified gadolinium oxide (Gd_2_O_3_) nanoparticles (SPG–DOX–Gd NPs) that function as an effective theranostic probe for cancer therapy and precise early diagnosis. The amount of conjugated DOX can be controlled by NP formulation during the preparation. In the HeLa cytotoxicity assay, SPG–DOX–Gd NPs inhibited cell growth as strongly as DOX *alone* because the surface SPG of the NPs was degraded by MMP-2 and/or -9 to liberate DOX, especially in HeLa cells. For imaging, SPG–DOX–Gd NPs were detected in the back tissues of mice by PA imaging after the subcutaneous administration of NPs. In addition, SPG–DOX–Gd NPs showed 3.28 and 1.23 times higher *r*_1_ values than those of Gd-DTPA and SPG–Gd NPs, respectively. After intravenous administration of SPG–DOX–Gd NPs to tumor-bearing mice, the NPs accumulated in the tumor tissue via the enhanced permeability and retention (EPR) effect. SPG–DOX–Gd NPs showed significant antitumor potential, as evidenced by suppressed tumor growth. The SI ratios (post 1 h/pre) derived from MR and PA images of the tumor site were 1.50 and 3.84 times higher than those after administration of DOX *alone* and PBS, respectively. Consequently, SPG–DOX–Gd NPs represent a promising theranostic probe that combines dual-modality imaging with antitumor treatment.

## Figures and Tables

**Table 1:** The *r*_1_, *r*_2_, and *r*_2_/*r*_1_ values of SPG–DOX–Gd NPs, SPG–Gd NPs, and Gd–DTPA (Magnevist^®^).

	SPG–DOX–Gd NPs	SPG–Gd NPs	Gd–DTPA
*r*_1_ (mM^−1^ s^−1^)	15.4	12.5	4.7
*r*_2_ (mM^−1^ s^−1^)	40.2	18.2	5.1
*r*_2_/*r*_1_	2.6	1.5	1.2

**Table 2:** The SI ratios in both MR and PA images of the tumor site on average.

	SPG–DOX–Gd NPs	DOX *alone*	PBS
MRI SI ratio (Day 0 Post 1 h/Pre)	1.50	1.00	1.00
PAI SI ratio (Day 0 Post 1 h/Pre)	3.84	1.66	1.43
PAI SI ratio (Day 11 Post 1 h/Pre)	1.52	1.09	1.06

## Data Availability

Data is contained within the article or Supplementary Materials.

## Supplementary Materials

The following supporting information can be downloaded at: https://www.mdpi.com/article/10.3390/nano16060343/s1, Figure S1: UV–Vis–NIR absorbance spectra of SPG–DOX–Gd NPs and controls; Figure S2: Particle size of synthesized nanoparticles. Data are presented as mean ± SD (*n* = 3); Figure S3: The surface potential of synthesized nanoparticles. Data are presented as mean ± SD (*n* = 3); Figure S4: The wash-out ratio of DOX in wash liquid of SPG–DOX–Gd NPs; Figure S5: Time-dependent release of DOX from SPG–DOX–Gd NPs in PBS with 10% FBS (pH 7.4) at 37 °C. Data are presented as mean ± SD (*n* = 3).

## References

[1] Kelkar S.S., Reineke T.M. (2011). Theranostics: Combining Imaging and Therapy. Bioconjug. Chem..

[2] Janib S.M., Moses A.S., MacKay J.A. (2010). Imaging and Drug Delivery Using Theranostic Nanoparticles. Adv. Drug Deliv. Rev..

[3] Lammers T., Aime S., Hennink W.E., Storm G., Kiessling F. (2011). Theranostic Nanomedicine. Acc. Chem. Res..

[4] Liang F., Zhang R., Zhong Q., Zhou Y., Xun X., Nezamzadeh-ejhieh A., Lu L., Ouyang Q., Huang Y. (2026). Tumor diagnosis and therapy system based on covalent organic framework (COF): From sensing detection to synergistic treatment. Colloids Surf. B Biointerfaces.

[5] Ehrhardt J.D., Güleç S. (2020). A Review of the History of Radioactive Iodine Theranostics: The Origin of Nuclear Ontology. Mol. Imaging Radionucl. Ther..

[6] Harris A.G., Vinik A.I., O’Dorisio T.M., O’Dorisio M.S. (2020). Radioligand Theranostics in the Management of Neuroendocrine Tumors. Pancreas.

[7] Jeitner T.M., Babich J.W., Kelly J.M. (2022). Advances in PSMA Theranostics. Transl. Oncol..

[8] Arnold C. (2022). Theranostics Could Be Big Business in Precision Oncology. Nat. Med..

[9] Bodei L., Herrmann K., Schöder H., Scott A.M., Lewis J.S. (2022). Radiotheranostics in oncology: Current challenges and emerging opportunities. Nat. Rev. Clin. Oncol..

[10] Zanzonico P. (2012). Principles of Nuclear Medicine Imaging: Planar, SPECT, PET, Multi-Modality, and Autoradiography Systems. Radiat. Res..

[11] Cherry S.R. (2009). Multimodality Imaging: Beyond PET/CT and SPECT/CT. Semin. Nucl. Med..

[12] Cai Y., Chen X., Si J., Mou X., Dong X. (2021). All-in-One Nanomedicine: Multifunctional Single-Component Nanoparticles for Cancer Theranostics. Small.

[13] Xiao D., Zhao L., Xie F., Fan S., Liu L., Li W., Cao R., Li S., Zhong W., Zhou X. (2021). A Bifunctional Molecule-Based Strategy for the Development of Theranostic Antibody-Drug Conjugate. Theranostics.

[14] Sievers E.L., Senter P.D. (2013). Antibody-Drug Conjugates in Cancer Therapy. Annu. Rev. Med..

[15] Beck A., Goetsch L., Dumontet C., Corvaïa N. (2017). Strategies and Challenges for the next Generation of Antibody-Drug Conjugates. Nat. Rev. Drug Discov..

[16] Manandhar S., Sjöholm E., Bobacka J., Rosenholm J.M., Bansal K.K. (2021). Polymer-Drug Conjugates as Nanotheranostic Agents. J. Nanotheranostics.

[17] Lim E.-K., Kim T., Paik S., Haam S., Huh Y.-M., Lee K. (2015). Nanomaterials for Theranostics: Recent Advances and Future Challenges. Chem. Rev..

[18] Maeda H. (2010). Tumor-Selective Delivery of Macromolecular Drugs via the EPR Effect: Background and Future Prospects. Bioconjug. Chem..

[19] Chow E.K.H., Ho D. (2013). Cancer Nanomedicine: From Drug Delivery to Imaging. Sci. Transl. Med..

[20] Callahan J., Kopečková P., Kopeček J. (2009). Intracellular Trafficking and Subcellular Distribution of a Large Array of HPMA Copolymers. Biomacromolecules.

[21] Rani S., Gupta U. (2020). HPMA-Based Polymeric Conjugates in Anticancer Therapeutics. Drug Discov. Today.

[22] Scott L.C., Yao J.C., Benson A.B., Thomas A.L., Falk S., Mena R.R., Picus J., Wright J., Mulcahy M.F., Ajani J.A. (2009). A Phase II Study of Pegylated-Camptothecin (Pegamotecan) in the Treatment of Locally Advanced and Metastatic Gastric and Gastro-Oesophageal Junction Adenocarcinoma. Cancer Chemother. Pharmacol..

[23] Eom T., Yoo W., Lee Y.D., Park J.H., Choe Y., Bang J., Kim S., Khan A. (2017). An Activatable Anticancer Polymer-Drug Conjugate Based on the Self-Immolative Azobenzene Motif. J. Mater. Chem. B.

[24] Khare V., Kour S., Alam N., Dubey R.D., Saneja A., Koul M., Gupta A.P., Singh D., Singh S.K., Saxena A.K. (2014). Synthesis, Characterization and Mechanistic-Insight into the Anti-Proliferative Potential of PLGA-Gemcitabine Conjugate. Int. J. Pharm..

[25] Kumar S., Meena V.K., Hazari P.P., Sharma R.K. (2017). PEG Coated and Doxorubicin Loaded Multimodal Gadolinium Oxide Nanoparticles for Simultaneous Drug Delivery and Imaging Applications. Int. J. Pharm..

[26] Elzoghby A.O. (2013). Gelatin-based nanoparticles as drug and gene delivery systems: Reviewing three decades of research. J. Control. Release.

[27] Wang Y., Xia H., Chen B., Wang Y. (2022). Rethinking Nanoparticulate Polymer–Drug Conjugates for Cancer Theranostics. Wiley Interdiscip. Rev. Nanomed. Nanobiotechnol..

[28] Madamsetty V.S., Mukherjee A., Mukherjee S. (2019). Recent Trends of the Bio-Inspired Nanoparticles in Cancer Theranostics. Front. Pharmacol..

[29] Mahdavi B., Shokrani P., Hejazi S.H., Talebi A., Taheri A. (2019). Doxorubicin-Loaded PVP Coated Gd_2_O_3_ NPs for Effective Chemoradiotherapy in Melanoma. J. Drug Deliv. Sci. Technol..

[30] Mishra S.K., Kannan S. (2017). Doxorubicin-Conjugated Bimetallic Silver-Gadolinium Nanoalloy for Multimodal MRI-CT-Optical Imaging and PH-Responsive Drug Release. ACS Biomater. Sci. Eng..

[31] El-Agamy S.E., Abdel-Aziz A.K., Esmat A., Azab S.S. (2019). Chemotherapy and Cognition: Comprehensive Review on Doxorubicin-Induced Chemobrain. Cancer Chemother. Pharmacol..

[32] Taymaz-Nikerel H., Karabekmez M.E., Eraslan S., Kırdar B. (2018). Doxorubicin Induces an Extensive Transcriptional and Metabolic Rewiring in Yeast Cells. Sci. Rep..

[33] Minotti G., Menna P., Salvatorelli E., Cairo G., Gianni L. (2004). Anthracyclines: Molecular Advances and Pharmacologie Developments in Antitumor Activity and Cardiotoxicity. Pharmacol. Rev..

[34] Ayen W.Y., Kumar N. (2012). A systematic study on lyophilization process of polymersomes for long-term storage using doxorubicin-loaded (PEG)3-PLA nanopolymersomes. Eur. J. Pharm. Sci..

[35] Choubey J., Bajpai A.K. (2010). Investigation on magnetically controlled delivery of doxorubicin from superparamagnetic nanocarriers of gelatin crosslinked with genipin. J. Mater. Sci. Mater. Med..

[36] Lee G.Y., Park K., Nam J.H., Kim S.Y., Byun Y. (2006). Anti-tumor and anti-metastatic effects of gelatin-doxorubicin and PEGylated gelatin-doxorubicin nanoparticles in SCC7 bearing mice. J. Drug Target..

[37] Sawaya R.E., Yamamoto M., Gokaslan Z.L., Wang S.W., Mohanam S., Fuller G.N., McCutcheon I.E., Stetler-Stevenson W.G., Nicolson G.L., Rao J.S. (1996). Expression and Localization of 72 KDa Type IV Collagenase (MMP-2) in Human Malignant Gliomas In Vivo. Clin. Exp. Metastasis.

[38] Leo E., Cameroni R., Forni F. (1999). Dynamic Dialysis for the Drug Release Evaluation from Doxorubicin. Int. J. Pharm..

[39] Kimura Y., Kurimoto T., Imai Y., Sugii H., Toshimitsu A., Matsuda T., Imai H., Yamada H., Kondo T. (2014). Novel Biocompatible Cobalt Oxide Nanoparticles for Use in Dual Photoacoustic and Magnetic Resonance Imaging. JSM Biotechnol. Bioeng..

[40] Sitharaman B., Kissell K.R., Hartman K.B., Tran L.A., Baikalov A., Rusakova I., Sun Y., Khant H.A., Ludtke S.J., Chiu W. (2005). Superparamagnetic Gadonanotubes Are High-Performance MRI Contrast Agents. Chem. Commun..

[41] Zou C., Wu B., Dong Y., Song Z., Zhao Y., Ni X., Yang Y., Liu Z. (2017). Biomedical Photoacoustics: Fundamentals, Instrumentation and Perspectives on Nanomedicine. Int. J. Nanomed..

[42] Tewey K.M., Rowe T.C., Yang L., Halligan B.D., Liu L.F. (1984). Adriamycin-Induced DNA Damage Mediated by Mammalian DNA Topoisomerase II. Science.

[43] Valluru K.S., Wilson K.E., Willmann J.K. (2016). Photoacoustic Imaging in Oncology: Translational Preclinical and Early Clinical Experience. Radiology.

[44] Kircher M.F., de la Zerda A., Jokerst J.V., Zavaleta C.L., Kempen P.J., Mittra E., Pitter K., Huang R., Campos C., Habte F. (2012). A Brain Tumor Molecular Imaging Strategy Using a New Triple-Modality MRI-Photoacoustic-Raman Nanoparticle. Nat. Med..

[45] Kimura Y., Kamisugi R., Narazaki M., Matsuda T., Tabata Y., Toshimitsu A., Kondo T. (2012). Size-Controlled and Biocompatible Gd_2_O_3_ Nanoparticles for Dual Photoacoustic and MR Imaging. Adv. Healthc. Mater..

[46] Wang X., Kimura Y., Miura R., Imai H., Kondo T. (2026). Cisplatin–incorporating Gelatin–coated Gadolinium Oxide Nanoparticles for Cancer Theranostics. iScience.

[47] Shah M., Bourner L., Ali S., Al-Enazy S., Youssef M.M., Fisler M., Rytting E. (2018). HPLC Method Development for Quantification of Doxorubicin in Cell Culture and Placental Perfusion Media. Separations.

[48] Zhu L., Kate P., Torchilin V.P. (2012). Matrix metalloprotease 2-responsive multifunctional liposomal nanocarrier for enhanced tumor targeting. ACS Nano.

[49] Zhu Q., Jia L., Gao Z., Wang C., Jiang H., Zhang J., Dong L. (2014). A Tumor Environment Responsive Doxorubicin-Loaded Nanoparticle for Targeted Cancer Therapy. Mol. Pharm..

[50] Smith A.M., Mancini M.C., Nie S. (2009). Bioimaging: Second window for in vivo imaging. Nat. Nanotechnol..

[51] Hemmer E., Benayas A., Légaré F., Vetrone F. (2016). Exploiting the biological windows: Current perspectives on fluorescent bioprobes emitting above 1000 nm. Nanoscale Horiz..

[52] Söderlind F., Pedersen H., Petoral R.M., Käll P.-O.O., Uvdal K.J. (2005). Synthesis and characterisation of Gd_2_O_3_ nanocrystals functionalised by organic acids. Colloid. Interface Sci..

[53] Ahmad M.Y., Ahmad M.W., Yue H., Ho S.L., Park J.A., Jung K.H., Cha H., Marasini S., Ghazanfari A., Liu S. (2020). In vivo positive magnetic resonance imaging applications of poly(methyl vinyl ether-alt-maleic acid)-coated ultra-small paramagnetic gadolinium oxide nanoparticles. Molecules.

[54] Marasini S., Yue H., Ghazanfari A., Ho S.L., Park J.A., Kim S., Cha H., Liu S., Tegafaw T., Ahmad M.Y. (2021). Polyaspartic acid-coated paramagnetic gadolinium oxide nanoparticles as a dual-modal t1 and t2 magnetic resonance imaging contrast agent. Appl. Sci..

[55] Long J.T., Cheang T.Y., Zhuo S.Y., Zeng R.F., Dai Q.S., Li H.P., Fang S. (2014). Anticancer drug-loaded multifunctional nanoparticles to enhance the chemotherapeutic efficacy in lung cancer metastasis. J. Nanobiotechnol..

[56] Zha Z., Zhang S., Deng Z., Li Y., Li C., Dai Z. (2013). Enzyme-Responsive Copper Sulphide Nanoparticles for Combined Photoacoustic Imaging, Tumor-Selective Chemotherapy and Photothermal Therapy. Chem. Commun..

[57] Zhang W., Liu L., Chen H., Hu K., Delahunty I., Gao S., Xie J. (2018). Surface impact on nanoparticle-based magnetic resonance imaging contrast agents. Theranostics.

[58] Li F., Liang Z., Liu J., Sun J., Hu X., Zhao M., Liu J., Bai R., Kim D., Sun X. (2019). Dynamically Reversible Iron Oxide Nanoparticle Assemblies for Targeted Amplification of T1-Weighted Magnetic Resonance Imaging of Tumors. Nano Lett..

[59] Wahsner J., Gale E.M., Rodríguez-Rodríguez A., Caravan P. (2019). Chemistry of MRI contrast agents: Current challenges and new frontiers. Chem. Rev..

[60] Jing L., Liang X., Li X., Yang Y., Dai Z. (2013). Covalent attachment of Mn-porphyrin onto doxorubicin-loaded poly(lactic acid) nanoparticles for potential magnetic resonance imaging and pH-sensitive drug delivery. Acta Biomater..

[61] Wang H., An L., Tao C., Ling Z., Lin J., Tian Q., Yang S. (2020). A smart theranostic platform for photoacoustic and magnetic resonance dual-imaging-guided photothermal-enhanced chemodynamic therapy. Nanoscale.

