# Supplementary material for: Biocompatible Gadolinium Oxide Nanoparticles Incorporated Doxorubicin Enables Magnetic Resonance and Photoacoustic Dual Imaging for Cancer Theranostics
Source: Nanomaterials (Basel). 2026 Mar 10;16(6):343. doi: 10.3390/nano16060343 (PMC13028626; doi:10.3390/nano16060343)
Supplement: Supplementary file 1 [file nanomaterials-16-00343-s001.zip › nanomaterials-4179107-supplementary.pdf]

Supporting Information

Biocompatible Gadolinium Oxide Nanoparticles Incorporated Doxorubicin Enables Magnetic Resonance and Photoacoustic Dual Imaging for Cancer Theranostics

Xingchen Wang [1], Yuta Imai [1], Yu Kimura [1,*], Risako Miura [1], Hirohiko Imai [2] and Teruyuki Kondo [1,*]

[1] Department of Energy and Hydrocarbon Chemistry, Graduate School of Engineering, Kyoto University, Kyoto 6158510, Japan; wang.xingchen.68s@st.kyoto-u.ac.jp (X.W.); yoshidanewyork@gmail.com (Y.I.); miura.risako.8x@kyoto-u.ac.jp (R.M.)

[2] Innovation Research Center for Quantum Medicine, Gifu University School of Medicine, Gifu 501-1194, Japan; imai.hirohiko.b1@f.gifu-u.ac.jp

* Correspondence: kimura.yuu.7m@kyoto-u.ac.jp (Y.K.); teruyuki@scl.kyoto-u.ac.jp (T.K.)

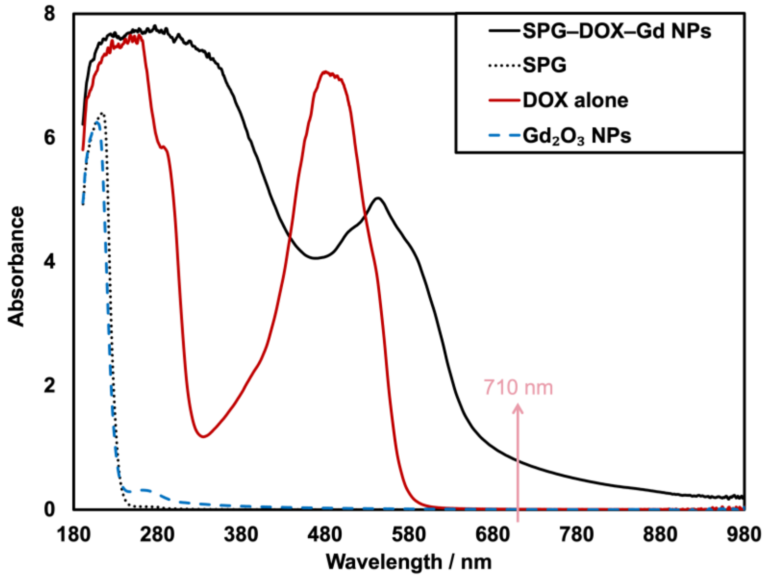

Figure S1. UV–Vis–NIR absorbance spectra of SPG–DOX–Gd NPs and controls.

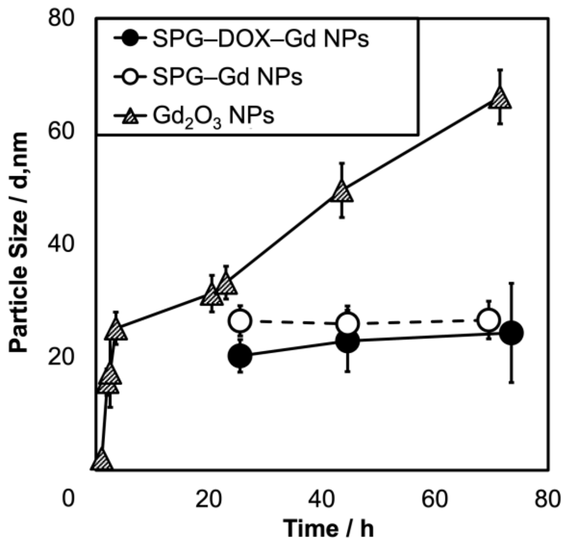

Figure S2. Particle size of synthesized nanoparticles. Data are presented as mean ± SD (n = 3).

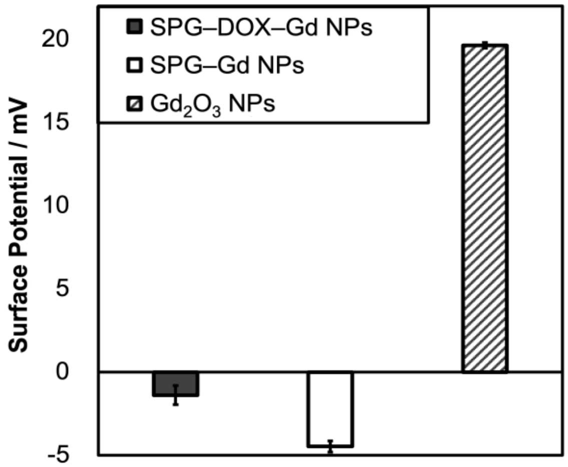

Figure S3. The surface potential of synthesized nanoparticles. Data are presented as mean ± SD (*n* = 3).

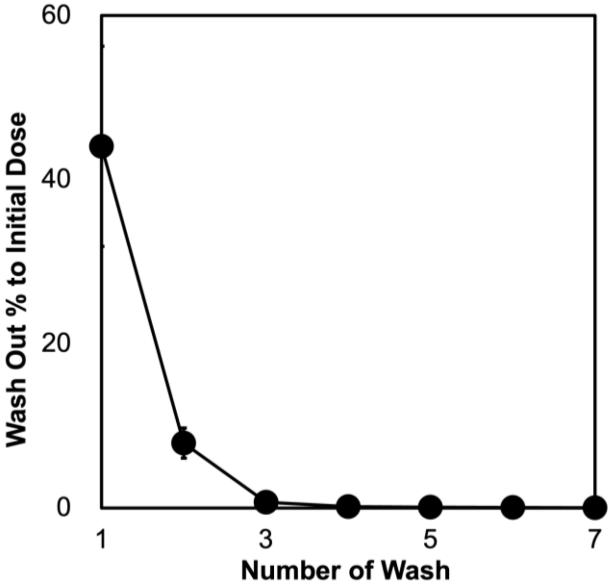

Figure S4. The wash-out ratio of DOX in wash liquid of SPG–DOX–Gd NPs.

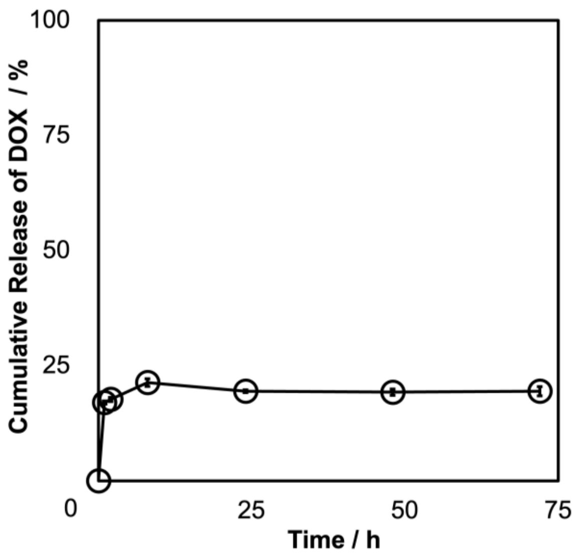

Figure S5. Time-dependent release of DOX from SPG–DOX–Gd NPs in PBS with 10% FBS (pH 7.4) at 37 °C. Data are presented as mean ± SD (*n* = 3).
